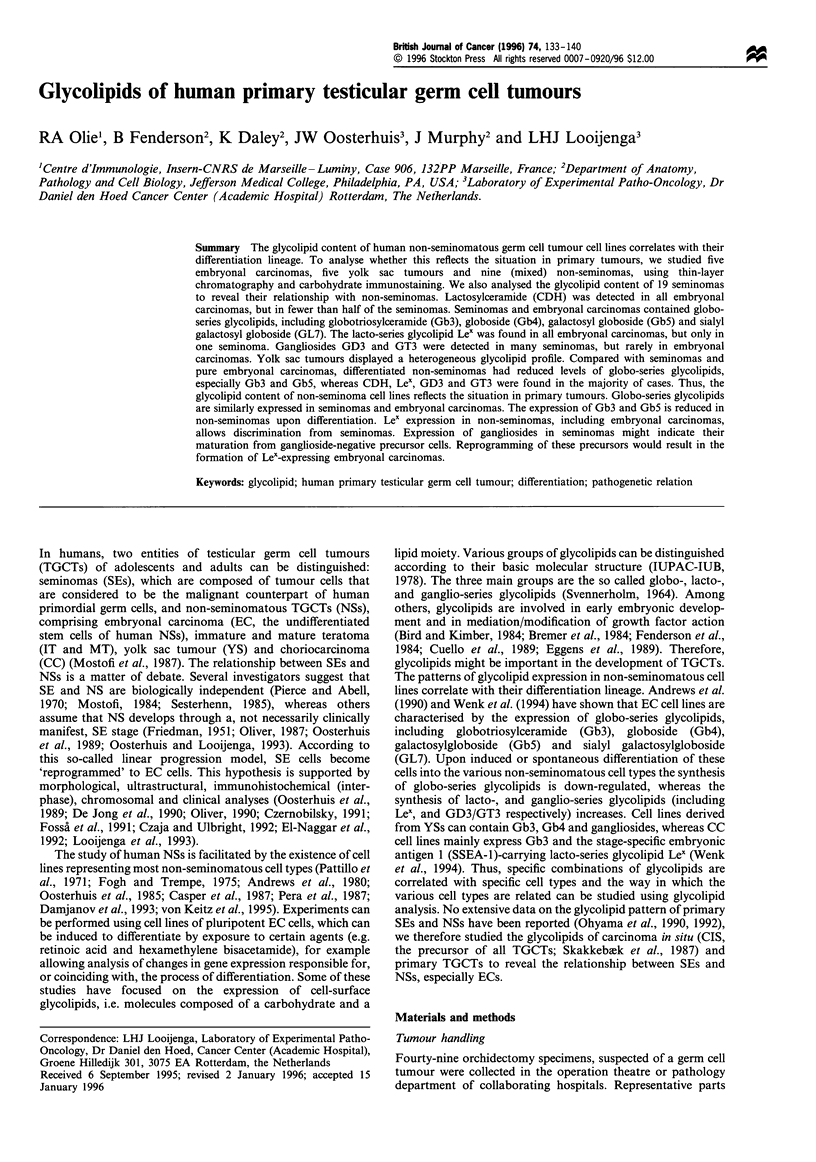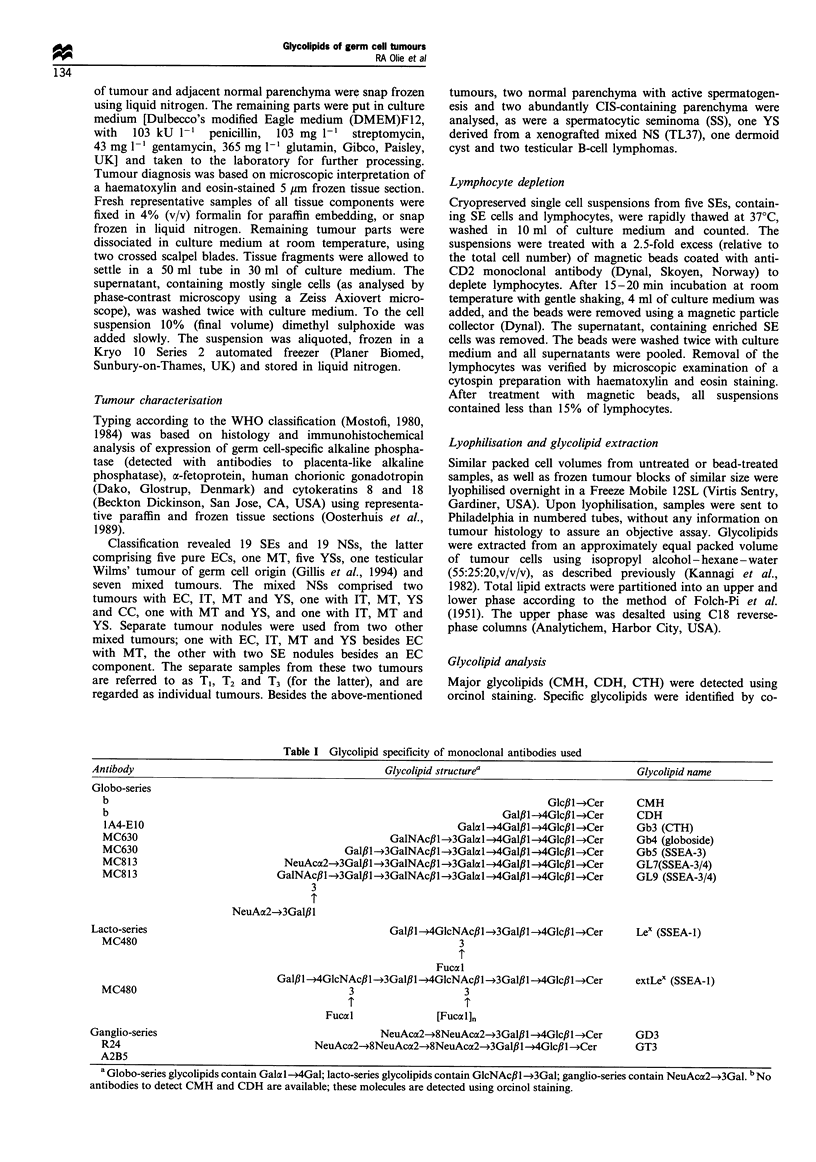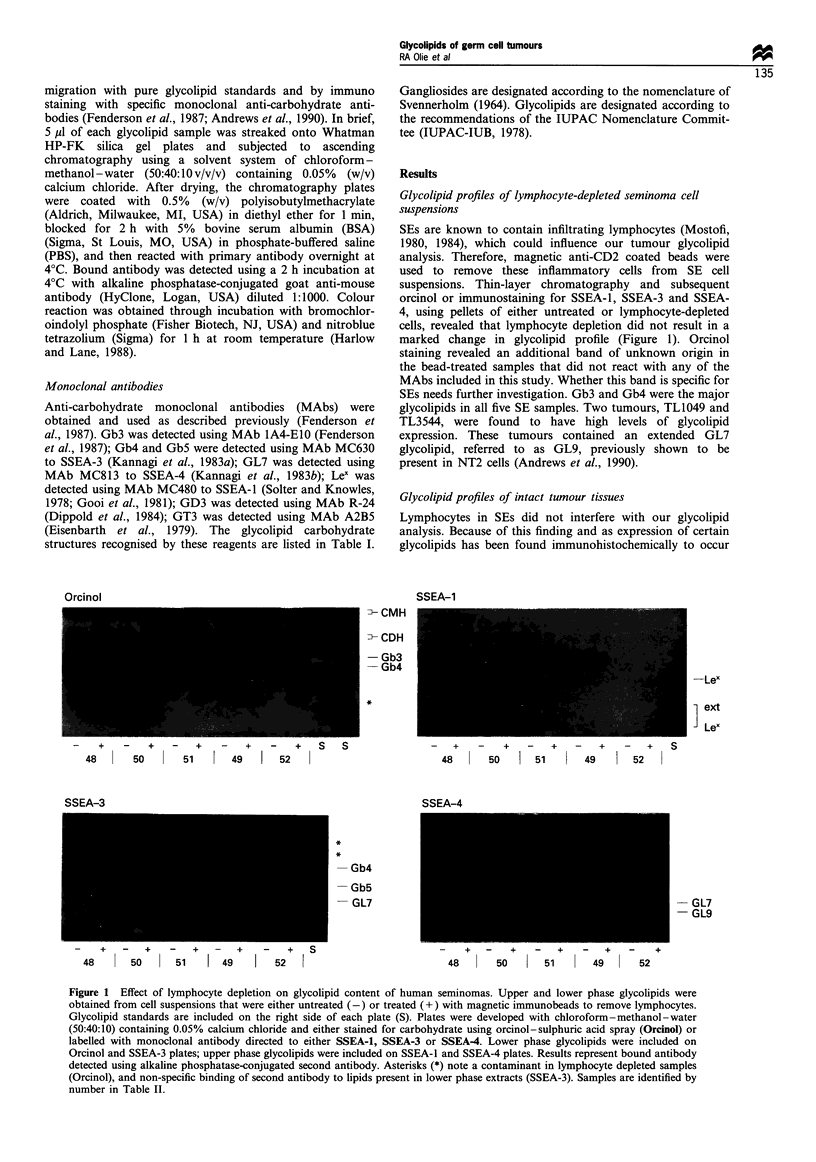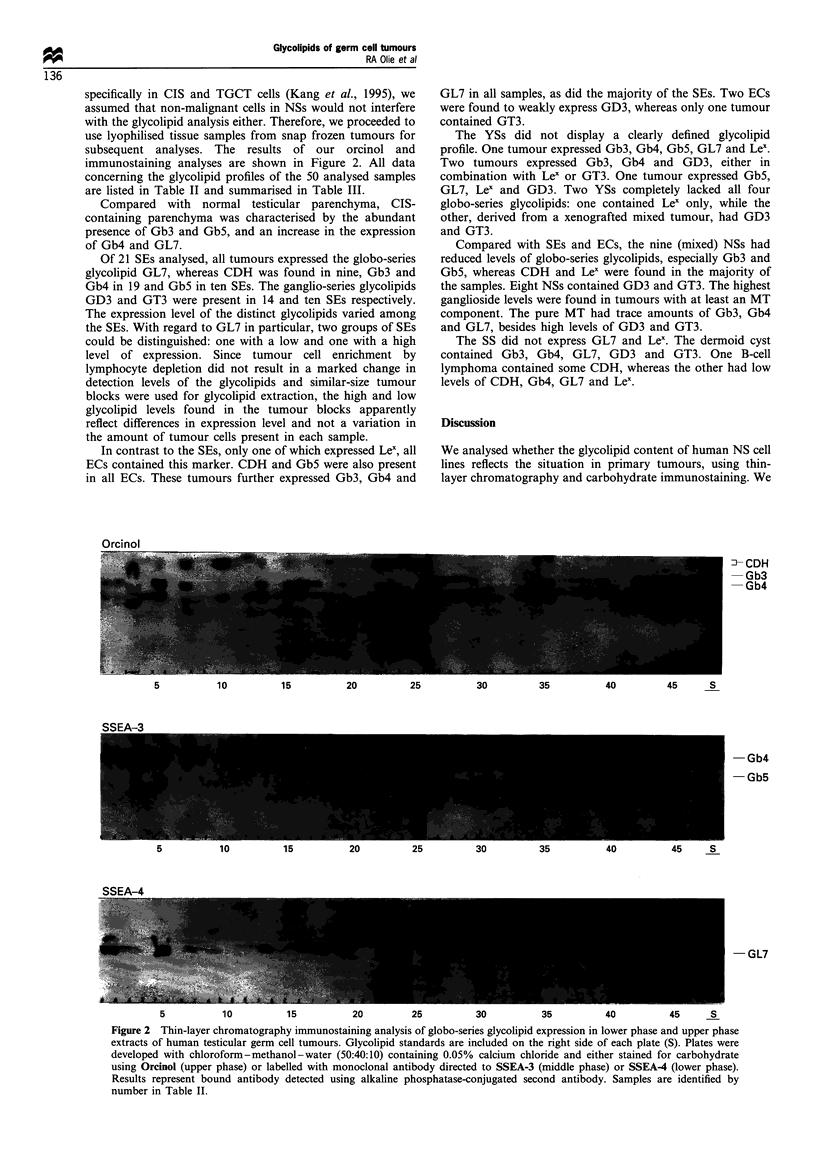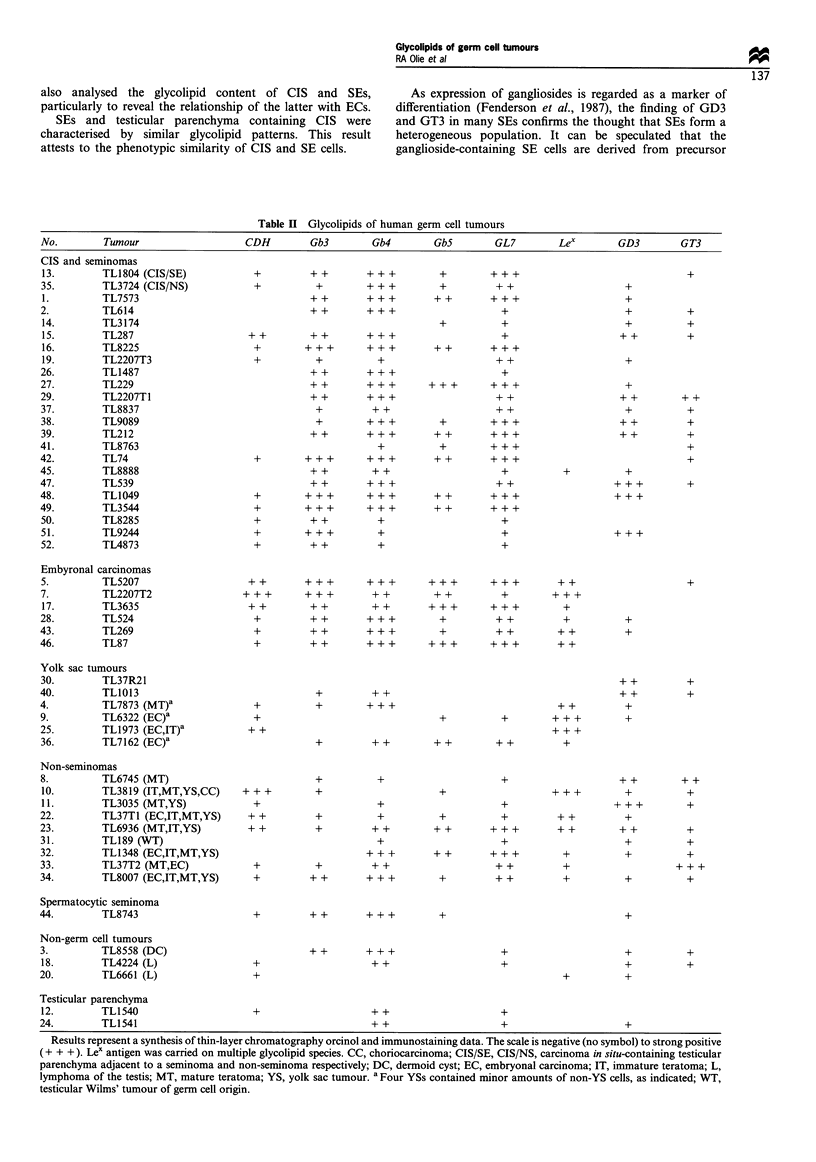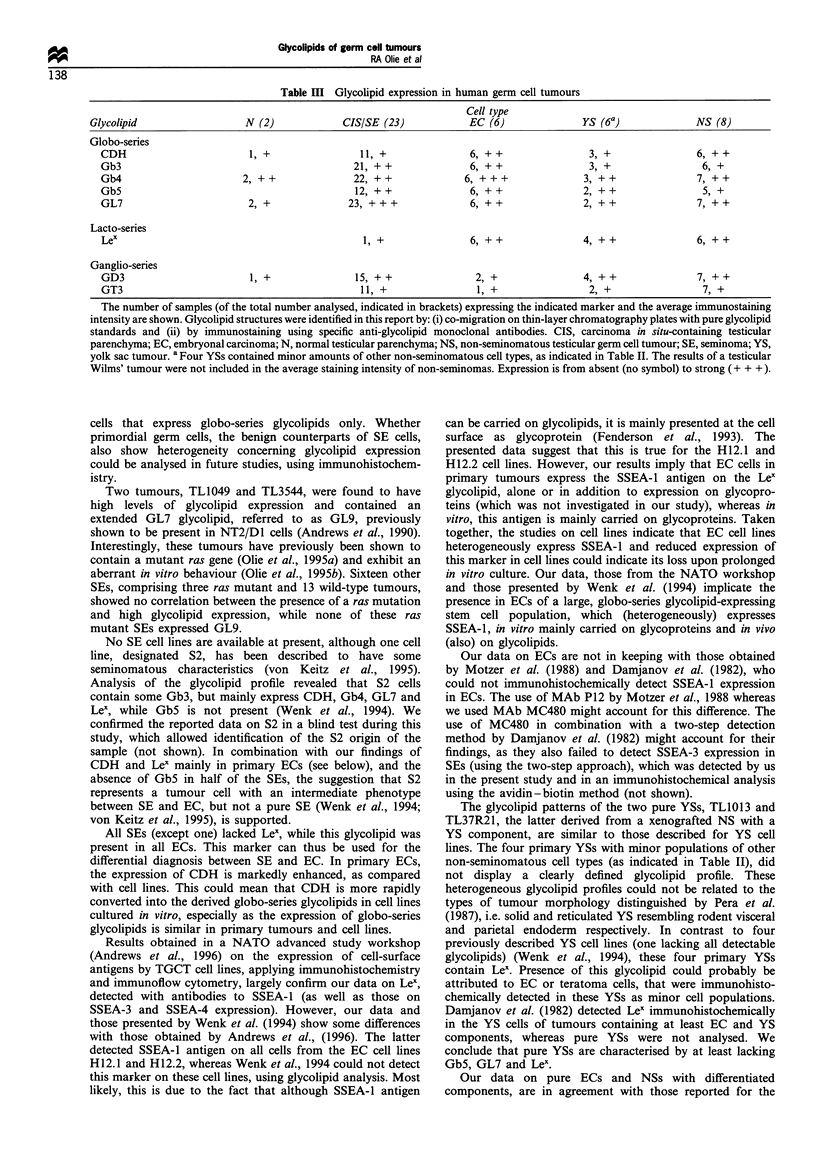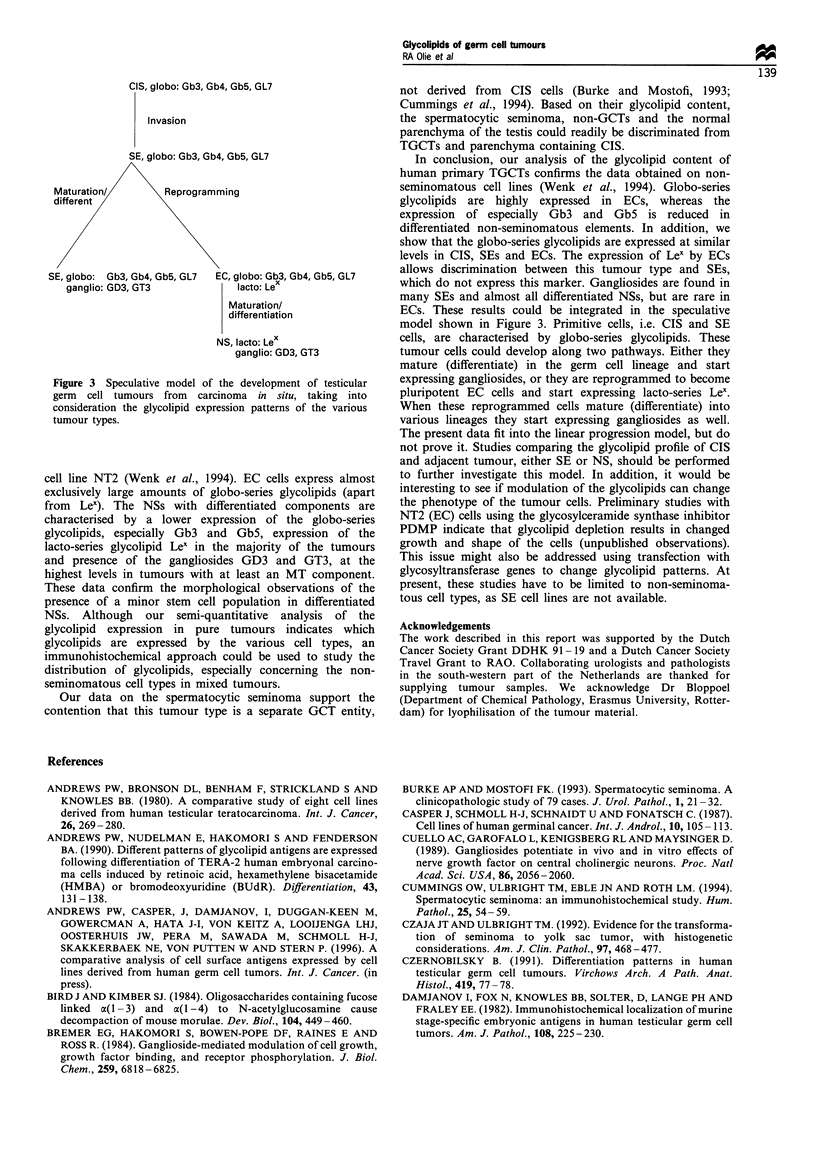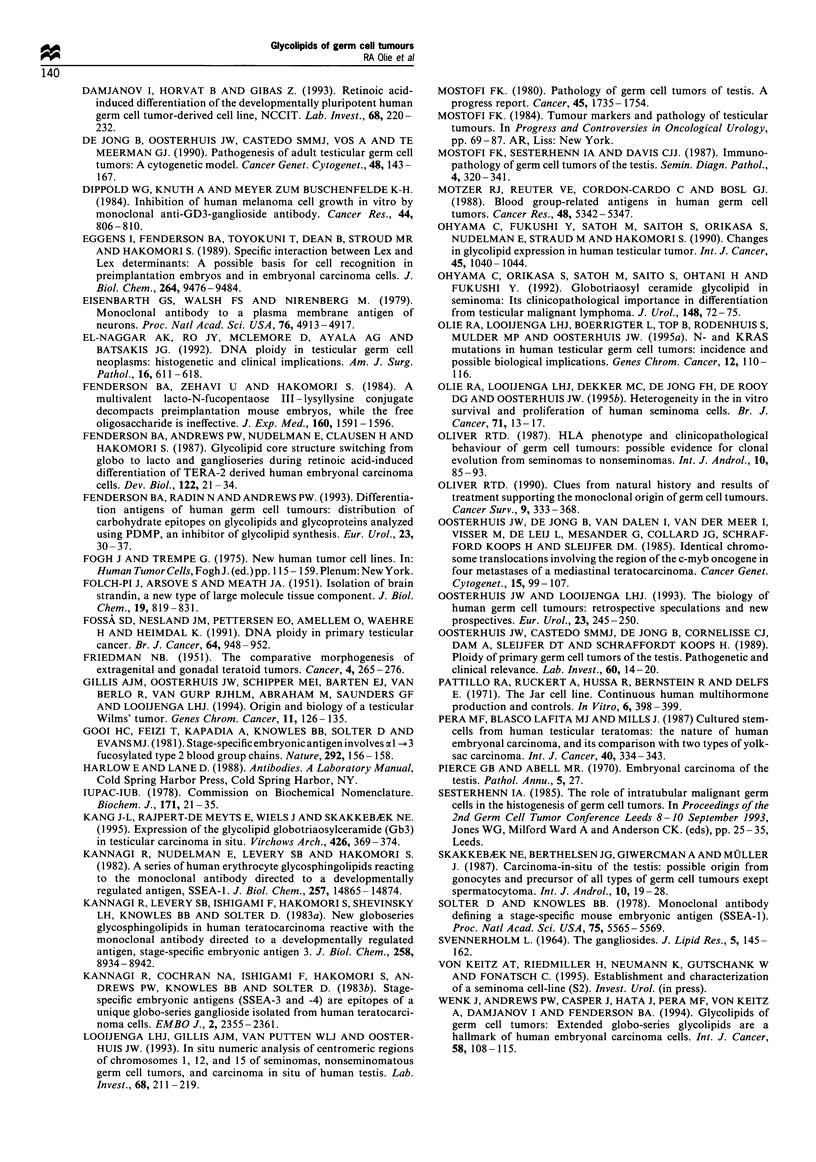# Glycolipids of human primary testicular germ cell tumours.

**DOI:** 10.1038/bjc.1996.328

**Published:** 1996-07

**Authors:** R. A. Olie, B. Fenderson, K. Daley, J. W. Oosterhuis, J. Murphy, L. H. Looijenga

**Affiliations:** Centre d'Immunologie, Insern-CNRS de Marseille-Luminy, France.

## Abstract

**Images:**


British Journal of Cancer (1996) 74, 133-140
© 1996 Stockton Press All rights reserved 0007-0920/96 $12.00

### Glycolipids of human primary testicular germ cell tumours

RA Olie[1], B Fenderson[2], K Daley[2], JW Oosterhuis[3], J Murphy[2] and LHJ Looijenga[3]

*[1]Centre d'Immunologie, Insern-CNRS de Marseille–Luminy, Case 906, 132PP Marseille, France; [2]Department of Anatomy, Pathology and Cell Biology, Jefferson Medical College, Philadelphia, PA, USA; [3]Laboratory of Experimental Patho-Oncology, Dr Daniel den Hoed Cancer Center (Academic Hospital) Rotterdam, The Netherlands.*

**Summary** The glycolipid content of human non-seminomatous germ cell tumour cell lines correlates with their differentiation lineage. To analyse whether this reflects the situation in primary tumours, we studied five embryonal carcinomas, five yolk sac tumours and nine (mixed) non-seminomas, using thin-layer chromatography and carbohydrate immunostaining. We also analysed the glycolipid content of 19 seminomas to reveal their relationship with non-seminomas. Lactosylceramide (CDH) was detected in all embryonal carcinomas, but in fewer than half of the seminomas. Seminomas and embryonal carcinomas contained globo-series glycolipids, including globotriosylceramide (Gb3), globoside (Gb4), galactosyl globoside (Gb5) and sialyl galactosyl globoside (GL7). The lacto-series glycolipid $Le^x$ was found in all embryonal carcinomas, but only in one seminoma. Gangliosides GD3 and GT3 were detected in many seminomas, but rarely in embryonal carcinomas. Yolk sac tumours displayed a heterogeneous glycolipid profile. Compared with seminomas and pure embryonal carcinomas, differentiated non-seminomas had reduced levels of globo-series glycolipids, especially Gb3 and Gb5, whereas CDH, $Le^x$, GD3 and GT3 were found in the majority of cases. Thus, the glycolipid content of non-seminoma cell lines reflects the situation in primary tumours. Globo-series glycolipids are similarly expressed in seminomas and embryonal carcinomas. The expression of Gb3 and Gb5 is reduced in non-seminomas upon differentiation. $Le^x$ expression in non-seminomas, including embryonal carcinomas, allows discrimination from seminomas. Expression of gangliosides in seminomas might indicate their maturation from ganglioside-negative precursor cells. Reprogramming of these precursors would result in the formation of $Le^x$-expressing embryonal carcinomas.

**Keywords:** glycolipid; human primary testicular germ cell tumour; differentiation; pathogenetic relation

In humans, two entities of testicular germ cell tumours (TGCTs) of adolescents and adults can be distinguished: seminomas (SEs), which are composed of tumour cells that are considered to be the malignant counterpart of human primordial germ cells, and non-seminomatous TGCTs (NSs), comprising embryonal carcinoma (EC, the undifferentiated stem cells of human NSs), immature and mature teratoma (IT and MT), yolk sac tumour (YS) and choriocarcinoma (CC) (Mostofi *et al.*, 1987). The relationship between SEs and NSs is a matter of debate. Several investigators suggest that SE and NS are biologically independent (Pierce and Abell, 1970; Mostofi, 1984; Sesterhenn, 1985), whereas others assume that NS develops through a, not necessarily clinically manifest, SE stage (Friedman, 1951; Oliver, 1987; Oosterhuis *et al.*, 1989; Oosterhuis and Looijenga, 1993). According to this so-called linear progression model, SE cells become 'reprogrammed' to EC cells. This hypothesis is supported by morphological, ultrastructural, immunohistochemical (interphase), chromosomal and clinical analyses (Oosterhuis *et al.*, 1989; De Jong *et al.*, 1990; Oliver, 1990; Czernobilsky, 1991; Fosså *et al.*, 1991; Czaja and Ulbright, 1992; El-Naggar *et al.*, 1992; Looijenga *et al.*, 1993).

The study of human NSs is facilitated by the existence of cell lines representing most non-seminomatous cell types (Pattillo *et al.*, 1971; Fogh and Trempe, 1975; Andrews *et al.*, 1980; Oosterhuis *et al.*, 1985; Casper *et al.*, 1987; Pera *et al.*, 1987; Damjanov *et al.*, 1993; von Keitz *et al.*, 1995). Experiments can be performed using cell lines of pluripotent EC cells, which can be induced to differentiate by exposure to certain agents (e.g. retinoic acid and hexamethylene bisacetamide), for example allowing analysis of changes in gene expression responsible for, or coinciding with, the process of differentiation. Some of these studies have focused on the expression of cell-surface glycolipids, i.e. molecules composed of a carbohydrate and a lipid moiety. Various groups of glycolipids can be distinguished according to their basic molecular structure (IUPAC-IUB, 1978). The three main groups are the so called globo-, lacto-, and ganglio-series glycolipids (Svennerholm, 1964). Among others, glycolipids are involved in early embryonic development and in mediation/modification of growth factor action (Bird and Kimber, 1984; Bremer *et al.*, 1984; Fenderson *et al.*, 1984; Cuello *et al.*, 1989; Eggens *et al.*, 1989). Therefore, glycolipids might be important in the development of TGCTs. The patterns of glycolipid expression in non-seminomatous cell lines correlate with their differentiation lineage. Andrews *et al.* (1990) and Wenk *et al.* (1994) have shown that EC cell lines are characterised by the expression of globo-series glycolipids, including globotriosylceramide (Gb3), globoside (Gb4), galactosylgloboside (Gb5) and sialyl galactosylgloboside (GL7). Upon induced or spontaneous differentiation of these cells into the various non-seminomatous cell types the synthesis of globo-series glycolipids is down-regulated, whereas the synthesis of lacto-, and ganglio-series glycolipids (including $Le^x$, and GD3/GT3 respectively) increases. Cell lines derived from YSs can contain Gb3, Gb4 and gangliosides, whereas CC cell lines mainly express Gb3 and the stage-specific embryonic antigen 1 (SSEA-1)-carrying lacto-series glycolipid $Le^x$ (Wenk *et al.*, 1994). Thus, specific combinations of glycolipids are correlated with specific cell types and the way in which the various cell types are related can be studied using glycolipid analysis. No extensive data on the glycolipid pattern of primary SEs and NSs have been reported (Ohyama *et al.*, 1990, 1992), we therefore studied the glycolipids of carcinoma *in situ* (CIS, the precursor of all TGCTs; Skakkebæk *et al.*, 1987) and primary TGCTs to reveal the relationship between SEs and NSs, especially ECs.

### Materials and methods

### *Tumour handling*

Fourty-nine orchidectomy specimens, suspected of a germ cell tumour were collected in the operation theatre or pathology department of collaborating hospitals. Representative parts

Correspondence: LHJ Looijenga, Laboratory of Experimental Patho-Oncology, Dr Daniel den Hoed, Cancer Center (Academic Hospital), Groene Hilledijk 301, 3075 EA Rotterdam, the Netherlands
Received 6 September 1995; revised 2 January 1996; accepted 15 January 1996

of tumour and adjacent normal parenchyma were snap frozen using liquid nitrogen. The remaining parts were put in culture medium [Dulbecco's modified Eagle medium (DMEM)F12, with 103 kU $l^{-1}$ penicillin, 103 mg $l^{-1}$ streptomycin, 43 mg $l^{-1}$ gentamycin, 365 mg $l^{-1}$ glutamin, Gibco, Paisley, UK] and taken to the laboratory for further processing. Tumour diagnosis was based on microscopic interpretation of a haematoxylin and eosin-stained 5 μm frozen tissue section. Fresh representative samples of all tissue components were fixed in 4% (v/v) formalin for paraffin embedding, or snap frozen in liquid nitrogen. Remaining tumour parts were dissociated in culture medium at room temperature, using two crossed scalpel blades. Tissue fragments were allowed to settle in a 50 ml tube in 30 ml of culture medium. The supernatant, containing mostly single cells (as analysed by phase-contrast microscopy using a Zeiss Axiovert microscope), was washed twice with culture medium. To the cell suspension 10% (final volume) dimethyl sulphoxide was added slowly. The suspension was aliquoted, frozen in a Kryo 10 Series 2 automated freezer (Planer Biomed, Sunbury-on-Thames, UK) and stored in liquid nitrogen.

### *Tumour characterisation*

Typing according to the WHO classification (Mostofi, 1980, 1984) was based on histology and immunohistochemical analysis of expression of germ cell-specific alkaline phosphatase (detected with antibodies to placenta-like alkaline phosphatase), α-fetoprotein, human chorionic gonadotropin (Dako, Glostrup, Denmark) and cytokeratins 8 and 18 (Beckton Dickinson, San Jose, CA, USA) using representative paraffin and frozen tissue sections (Oosterhuis *et al.*, 1989).

Classification revealed 19 SEs and 19 NSs, the latter comprising five pure ECs, one MT, five YSs, one testicular Wilms' tumour of germ cell origin (Gillis *et al.*, 1994) and seven mixed tumours. The mixed NSs comprised two tumours with EC, IT, MT and YS, one with IT, MT, YS and CC, one with MT and YS, and one with IT, MT and YS. Separate tumour nodules were used from two other mixed tumours; one with EC, IT, MT and YS besides EC with MT, the other with two SE nodules besides an EC component. The separate samples from these two tumours are referred to as $T_1$, $T_2$ and $T_3$ (for the latter), and are regarded as individual tumours. Besides the above-mentioned tumours, two normal parenchyma with active spermatogenesis and two abundantly CIS-containing parenchyma were analysed, as were a spermatocytic seminoma (SS), one YS derived from a xenografted mixed NS (TL37), one dermoid cyst and two testicular B-cell lymphomas.

### *Lymphocyte depletion*

Cryopreserved single cell suspensions from five SEs, containing SE cells and lymphocytes, were rapidly thawed at 37°C, washed in 10 ml of culture medium and counted. The suspensions were treated with a 2.5-fold excess (relative to the total cell number) of magnetic beads coated with anti-CD2 monoclonal antibody (Dynal, Skoyen, Norway) to deplete lymphocytes. After 15–20 min incubation at room temperature with gentle shaking, 4 ml of culture medium was added, and the beads were removed using a magnetic particle collector (Dynal). The supernatant, containing enriched SE cells was removed. The beads were washed twice with culture medium and all supernatants were pooled. Removal of the lymphocytes was verified by microscopic examination of a cytospin preparation with haematoxylin and eosin staining. After treatment with magnetic beads, all suspensions contained less than 15% of lymphocytes.

### *Lyophilisation and glycolipid extraction*

Similar packed cell volumes from untreated or bead-treated samples, as well as frozen tumour blocks of similar size were lyophilised overnight in a Freeze Mobile 12SL (Virtis Sentry, Gardiner, USA). Upon lyophilisation, samples were sent to Philadelphia in numbered tubes, without any information on tumour histology to assure an objective assay. Glycolipids were extracted from an approximately equal packed volume of tumour cells using isopropyl alcohol–hexane–water (55:25:20,v/v/v), as described previously (Kannagi *et al.*, 1982). Total lipid extracts were partitioned into an upper and lower phase according to the method of Folch-Pi *et al.* (1951). The upper phase was desalted using C18 reverse-phase columns (Analytichem, Harbor City, USA).

### *Glycolipid analysis*

Major glycolipids (CMH, CDH, CTH) were detected using orcinol staining. Specific glycolipids were identified by co-

**Table I** Glycolipid specificity of monoclonal antibodies used

| *Antibody* | *Glycolipid structure*[a] | *Glycolipid name* |
|---|---|---|
| Globo-series | | |
| b | Glcβ1→Cer | CMH |
| b | Galβ1→4Glcβ1→Cer | CDH |
| 1A4-E10 | Galα1→4Galβ1→4Glcβ1→Cer | Gb3 (CTH) |
| MC630 | GalNAcβ1→3Galα1→4Galβ1→4Glcβ1→Cer | Gb4 (globoside) |
| MC630 | Galβ1→3GalNAcβ1→3Galα1→4Galβ1→4Glcβ1→Cer | Gb5 (SSEA-3) |
| MC813 | NeuAcα2→3Galβ1→3GalNAcβ1→3Galα1→4Galβ1→4Glcβ1→Cer | GL7(SSEA-3/4) |
| MC813 | GalNAcβ1→3Galβ1→3GalNAcβ1→3Galα1→4Galβ1→4Glcβ1→Cer<br>3<br>↑<br>NeuAα2→3Galβ1 | GL9 (SSEA-3/4) |
| Lacto-series | | |
| MC480 | Galβ1→4GlcNAcβ1→3Galβ1→4Glcβ1→Cer<br>3<br>↑<br>Fucα1 | $Le^x$ (SSEA-1) |
| MC480 | Galβ1→4GlcNAcβ1→3Galβ1→4GlcNAcβ1→3Galβ1→4Glcβ1→Cer<br>3 3<br>↑ ↑<br>Fucα1 $[Fuc\alpha1]_n$ | ext$Le^x$ (SSEA-1) |
| Ganglio-series | | |
| R24 | NeuAcα2→8NeuAcα2→3Galβ1→4Glcβ1→Cer | GD3 |
| A2B5 | NeuAcα2→8NeuAcα2→8NeuAcα2→3Galβ1→4Glcβ1→Cer | GT3 |

[a] Globo-series glycolipids contain Galα1→4Gal; lacto-series glycolipids contain GlcNAcβ1→3Gal; ganglio-series contain NeuAcα2→3Gal. [b] No antibodies to detect CMH and CDH are available; these molecules are detected using orcinol staining.

migration with pure glycolipid standards and by immuno staining with specific monoclonal anti-carbohydrate antibodies (Fenderson et al., 1987; Andrews et al., 1990). In brief, 5 μl of each glycolipid sample was streaked onto Whatman HP-FK silica gel plates and subjected to ascending chromatography using a solvent system of chloroform–methanol–water (50:40:10 v/v/v) containing 0.05% (w/v) calcium chloride. After drying, the chromatography plates were coated with 0.5% (w/v) polyisobutylmethacrylate (Aldrich, Milwaukee, MI, USA) in diethyl ether for 1 min, blocked for 2 h with 5% bovine serum albumin (BSA) (Sigma, St Louis, MO, USA) in phosphate-buffered saline (PBS), and then reacted with primary antibody overnight at 4°C. Bound antibody was detected using a 2 h incubation at 4°C with alkaline phosphatase-conjugated goat anti-mouse antibody (HyClone, Logan, USA) diluted 1:1000. Colour reaction was obtained through incubation with bromochloroindolyl phosphate (Fisher Biotech, NJ, USA) and nitroblue tetrazolium (Sigma) for 1 h at room temperature (Harlow and Lane, 1988).

### *Monoclonal antibodies*

Anti-carbohydrate monoclonal antibodies (MAbs) were obtained and used as described previously (Fenderson et al., 1987). Gb3 was detected using MAb 1A4-E10 (Fenderson et al., 1987); Gb4 and Gb5 were detected using MAb MC630 to SSEA-3 (Kannagi et al., 1983a); GL7 was detected using MAb MC813 to SSEA-4 (Kannagi et al., 1983b); $Le^x$ was detected using MAb MC480 to SSEA-1 (Solter and Knowles, 1978; Gooi et al., 1981); GD3 was detected using MAb R-24 (Dippold et al., 1984); GT3 was detected using MAb A2B5 (Eisenbarth et al., 1979). The glycolipid carbohydrate structures recognised by these reagents are listed in Table I.

Gangliosides are designated according to the nomenclature of Svennerholm (1964). Glycolipids are designated according to the recommendations of the IUPAC Nomenclature Committee (IUPAC-IUB, 1978).

### Results

### *Glycolipid profiles of lymphocyte-depleted seminoma cell suspensions*

SEs are known to contain infiltrating lymphocytes (Mostofi, 1980, 1984), which could influence our tumour glycolipid analysis. Therefore, magnetic anti-CD2 coated beads were used to remove these inflammatory cells from SE cell suspensions. Thin-layer chromatography and subsequent orcinol or immunostaining for SSEA-1, SSEA-3 and SSEA-4, using pellets of either untreated or lymphocyte-depleted cells, revealed that lymphocyte depletion did not result in a marked change in glycolipid profile (Figure 1). Orcinol staining revealed an additional band of unknown origin in the bead-treated samples that did not react with any of the MAbs included in this study. Whether this band is specific for SEs needs further investigation. Gb3 and Gb4 were the major glycolipids in all five SE samples. Two tumours, TL1049 and TL3544, were found to have high levels of glycolipid expression. These tumours contained an extended GL7 glycolipid, referred to as GL9, previously shown to be present in NT2 cells (Andrews et al., 1990).

### *Glycolipid profiles of intact tumour tissues*

Lymphocytes in SEs did not interfere with our glycolipid analysis. Because of this finding and as expression of certain glycolipids has been found immunohistochemically to occur

**Figure 1** Effect of lymphocyte depletion on glycolipid content of human seminomas. Upper and lower phase glycolipids were obtained from cell suspensions that were either untreated (−) or treated (+) with magnetic immunobeads to remove lymphocytes. Glycolipid standards are included on the right side of each plate (S). Plates were developed with chloroform–methanol–water (50:40:10) containing 0.05% calcium chloride and either stained for carbohydrate using orcinol–sulphuric acid spray (**Orcinol**) or labelled with monoclonal antibody directed to either **SSEA-1, SSEA-3** or **SSEA-4**. Lower phase glycolipids were included on Orcinol and SSEA-3 plates; upper phase glycolipids were included on SSEA-1 and SSEA-4 plates. Results represent bound antibody detected using alkaline phosphatase-conjugated second antibody. Asterisks (*) note a contaminant in lymphocyte depleted samples (Orcinol), and non-specific binding of second antibody to lipids present in lower phase extracts (SSEA-3). Samples are identified by number in Table II.

specifically in CIS and TGCT cells (Kang *et al*., 1995), we assumed that non-malignant cells in NSs would not interfere with the glycolipid analysis either. Therefore, we proceeded to use lyophilised tissue samples from snap frozen tumours for subsequent analyses. The results of our orcinol and immunostaining analyses are shown in Figure 2. All data concerning the glycolipid profiles of the 50 analysed samples are listed in Table II and summarised in Table III.

Compared with normal testicular parenchyma, CIS-containing parenchyma was characterised by the abundant presence of Gb3 and Gb5, and an increase in the expression of Gb4 and GL7.

Of 21 SEs analysed, all tumours expressed the globo-series glycolipid GL7, whereas CDH was found in nine, Gb3 and Gb4 in 19 and Gb5 in ten SEs. The ganglio-series glycolipids GD3 and GT3 were present in 14 and ten SEs respectively. The expression level of the distinct glycolipids varied among the SEs. With regard to GL7 in particular, two groups of SEs could be distinguished: one with a low and one with a high level of expression. Since tumour cell enrichment by lymphocyte depletion did not result in a marked change in detection levels of the glycolipids and similar-size tumour blocks were used for glycolipid extraction, the high and low glycolipid levels found in the tumour blocks apparently reflect differences in expression level and not a variation in the amount of tumour cells present in each sample.

In contrast to the SEs, only one of which expressed $Le^x$, all ECs contained this marker. CDH and Gb5 were also present in all ECs. These tumours further expressed Gb3, Gb4 and GL7 in all samples, as did the majority of the SEs. Two ECs were found to weakly express GD3, whereas only one tumour contained GT3.

The YSs did not display a clearly defined glycolipid profile. One tumour expressed Gb3, Gb4, Gb5, GL7 and $Le^x$. Two tumours expressed Gb3, Gb4 and GD3, either in combination with $Le^x$ or GT3. One tumour expressed Gb5, GL7, $Le^x$ and GD3. Two YSs completely lacked all four globo-series glycolipids: one contained $Le^x$ only, while the other, derived from a xenografted mixed tumour, had GD3 and GT3.

Compared with SEs and ECs, the nine (mixed) NSs had reduced levels of globo-series glycolipids, especially Gb3 and Gb5, whereas CDH and $Le^x$ were found in the majority of the samples. Eight NSs contained GD3 and GT3. The highest ganglioside levels were found in tumours with at least an MT component. The pure MT had trace amounts of Gb3, Gb4 and GL7, besides high levels of GD3 and GT3.

The SS did not express GL7 and $Le^x$. The dermoid cyst contained Gb3, Gb4, GL7, GD3 and GT3. One B-cell lymphoma contained some CDH, whereas the other had low levels of CDH, Gb4, GL7 and $Le^x$.

### Discussion

We analysed whether the glycolipid content of human NS cell lines reflects the situation in primary tumours, using thin-layer chromatography and carbohydrate immunostaining. We

**Figure 2** Thin-layer chromatography immunostaining analysis of globo-series glycolipid expression in lower phase and upper phase extracts of human testicular germ cell tumours. Glycolipid standards are included on the right side of each plate (S). Plates were developed with chloroform–methanol–water (50:40:10) containing 0.05% calcium chloride and either stained for carbohydrate using **Orcinol** (upper phase) or labelled with monoclonal antibody directed to **SSEA-3** (middle phase) or **SSEA-4** (lower phase). Results represent bound antibody detected using alkaline phosphatase-conjugated second antibody. Samples are identified by number in Table II.

also analysed the glycolipid content of CIS and SEs, particularly to reveal the relationship of the latter with ECs.

SEs and testicular parenchyma containing CIS were characterised by similar glycolipid patterns. This result attests to the phenotypic similarity of CIS and SE cells.

As expression of gangliosides is regarded as a marker of differentiation (Fenderson *et al.*, 1987), the finding of GD3 and GT3 in many SEs confirms the thought that SEs form a heterogeneous population. It can be speculated that the ganglioside-containing SE cells are derived from precursor

**Table II** Glycolipids of human germ cell tumours

| *No.* | *Tumour* | *CDH* | *Gb3* | *Gb4* | *Gb5* | *GL7* | *Le*$^x$ | *GD3* | *GT3* |
|---|---|---|---|---|---|---|---|---|---|
| CIS and seminomas | | | | | | | | | |
| 13. | TL1804 (CIS/SE) | + | + + | + + + | + | + + + | | | + |
| 35. | TL3724 (CIS/NS) | + | + | + + + | + | + + | | + | |
| 1. | TL7573 | | + + | + + + | + + | + + + | | + | |
| 2. | TL614 | | + + | + + + | | + | | + | + |
| 14. | TL3174 | | | | + | + | | + | + |
| 15. | TL287 | + + | + + | + + + | | + | | + + | + |
| 16. | TL8225 | + | + + + | + + + | + + | + + + | | | |
| 19. | TL2207T3 | + | + | + | | + + | | + | |
| 26. | TL1487 | | + + | + + + | | + | | | |
| 27. | TL229 | | + + | + + + | + + + | + + + | | + | |
| 29. | TL2207T1 | | + + | + + + | | + + | | + + | + + |
| 37. | TL8837 | | + | + + | | + + | | + | + |
| 38. | TL9089 | | + | + + + | + | + + + | | + + | + |
| 39. | TL212 | | + + | + + + | + + | + + + | | + + | + |
| 41. | TL8763 | | | + | + | + + + | | | + |
| 42. | TL74 | + | + + + | + + + | + + | + + + | | | + |
| 45. | TL8888 | | + + | + + | | + | + | + | |
| 47. | TL539 | | + + | + + + | | + + | | + + + | + |
| 48. | TL1049 | + | + + + | + + + | + + | + + + | | + + + | |
| 49. | TL3544 | + | + + + | + + + | + + | + + + | | | |
| 50. | TL8285 | + | + + | + | | + | | | |
| 51. | TL9244 | + | + + + | + | | + | | + + + | |
| 52. | TL4873 | + | + + | + | | + | | | |
| Embyronal carcinomas | | | | | | | | | |
| 5. | TL5207 | + + | + + + | + + + | + + + | + + + | + + | | + |
| 7. | TL2207T2 | + + + | + + + | + + | + + | + | + + + | | |
| 17. | TL3635 | + + | + + | + + | + + + | + + + | + | | |
| 28. | TL524 | + | + + | + + + | + | + + | + | + | |
| 43. | TL269 | + | + + | + + + | + | + + | + + | + | |
| 46. | TL87 | + | + + | + + + | + + + | + + + | + + | | |
| Yolk sac tumours | | | | | | | | | |
| 30. | TL37R21 | | | | | | | + + | + |
| 40. | TL1013 | | + | + + | | | | + + | + |
| 4. | TL7873 (MT)[a] | + | + | + + + | | | + + | + | |
| 9. | TL6322 (EC)[a] | + | | | + | + | + + + | + | |
| 25. | TL1973 (EC,IT)[a] | + + | | | | | + + + | | |
| 36. | TL7162 (EC)[a] | | + | + + | + + | + + | + | | |
| Non-seminomas | | | | | | | | | |
| 8. | TL6745 (MT) | | + | + | | + | | + + | + + |
| 10. | TL3819 (IT,MT,YS,CC) | + + + | + | | + | | + + + | + | + |
| 11. | TL3035 (MT,YS) | + | | + | | + | | + + + | + |
| 22. | TL37T1 (EC,IT,MT,YS) | + + | + | + | + | + | + + | + | |
| 23. | TL6936 (MT,IT,YS) | + + | + | + + | + + | + + + | + + | + + | + |
| 31. | TL189 (WT) | | | + | | + | | + | + |
| 32. | TL1348 (EC,IT,MT,YS) | | | + + + | + + | + + + | + | + | + |
| 33. | TL37T2 (MT,EC) | + | + | + + | | + + | + | | + + + |
| 34. | TL8007 (EC,IT,MT,YS) | + | + + | + + + | + | + + | + | + | + |
| Spermatocytic seminoma | | | | | | | | | |
| 44. | TL8743 | + | + + | + + + | + | | | + | |
| Non-germ cell tumours | | | | | | | | | |
| 3. | TL8558 (DC) | | + + | + + + | | + | | + | + |
| 18. | TL4224 (L) | + | | + + | | + | | + | + |
| 20. | TL6661 (L) | + | | | | | + | + | |
| Testicular parenchyma | | | | | | | | | |
| 12. | TL1540 | + | | + + | | + | | | |
| 24. | TL1541 | | | + + | | + | | + | |

Results represent a synthesis of thin-layer chromatography orcinol and immunostaining data. The scale is negative (no symbol) to strong positive (+ + +). Le$^x$ antigen was carried on multiple glycolipid species. CC, choriocarcinoma; CIS/SE, CIS/NS, carcinoma *in situ*-containing testicular parenchyma adjacent to a seminoma and non-seminoma respectively; DC, dermoid cyst; EC, embryonal carcinoma; IT, immature teratoma; L, lymphoma of the testis; MT, mature teratoma; YS, yolk sac tumour. [a] Four YSs contained minor amounts of non-YS cells, as indicated; WT, testicular Wilms' tumour of germ cell origin.

**Table III** Glycolipid expression in human germ cell tumours

| Glycolipid | N (2) | CIS/SE (23) | Cell type EC (6) | YS (6[a]) | NS (8) |
|---|---|---|---|---|---|
| Globo-series | | | | | |
| CDH | 1, + | 11, + | 6, ++ | 3, + | 6, ++ |
| Gb3 | | 21, ++ | 6, ++ | 3, + | 6, + |
| Gb4 | 2, ++ | 22, ++ | 6, +++ | 3, ++ | 7, ++ |
| Gb5 | | 12, ++ | 6, ++ | 2, ++ | 5, + |
| GL7 | 2, + | 23, +++ | 6, ++ | 2, ++ | 7, ++ |
| Lacto-series | | | | | |
| $Le^x$ | | 1, + | 6, ++ | 4, ++ | 6, ++ |
| Ganglio-series | | | | | |
| GD3 | 1, + | 15, ++ | 2, + | 4, ++ | 7, ++ |
| GT3 | | 11, + | 1, + | 2, + | 7, + |

The number of samples (of the total number analysed, indicated in brackets) expressing the indicated marker and the average immunostaining intensity are shown. Glycolipid structures were identified in this report by: (i) co-migration on thin-layer chromatography plates with pure glycolipid standards and (ii) by immunostaining using specific anti-glycolipid monoclonal antibodies. CIS, carcinoma *in situ*-containing testicular parenchyma; EC, embryonal carcinoma; N, normal testicular parenchyma; NS, non-seminomatous testicular germ cell tumour; SE, seminoma; YS, yolk sac tumour. [a] Four YSs contained minor amounts of other non-seminomatous cell types, as indicated in Table II. The results of a testicular Wilms' tumour were not included in the average staining intensity of non-seminomas. Expression is from absent (no symbol) to strong (+++).

cells that express globo-series glycolipids only. Whether primordial germ cells, the benign counterparts of SE cells, also show heterogeneity concerning glycolipid expression could be analysed in future studies, using immunohistochemistry.

Two tumours, TL1049 and TL3544, were found to have high levels of glycolipid expression and contained an extended GL7 glycolipid, referred to as GL9, previously shown to be present in NT2/D1 cells (Andrews *et al.*, 1990). Interestingly, these tumours have previously been shown to contain a mutant *ras* gene (Olie *et al.*, 1995*a*) and exhibit an aberrant *in vitro* behaviour (Olie *et al.*, 1995*b*). Sixteen other SEs, comprising three *ras* mutant and 13 wild-type tumours, showed no correlation between the presence of a *ras* mutation and high glycolipid expression, while none of these *ras* mutant SEs expressed GL9.

No SE cell lines are available at present, although one cell line, designated S2, has been described to have some seminomatous characteristics (von Keitz *et al.*, 1995). Analysis of the glycolipid profile revealed that S2 cells contain some Gb3, but mainly express CDH, Gb4, GL7 and $Le^x$, while Gb5 is not present (Wenk *et al.*, 1994). We confirmed the reported data on S2 in a blind test during this study, which allowed identification of the S2 origin of the sample (not shown). In combination with our findings of CDH and $Le^x$ mainly in primary ECs (see below), and the absence of Gb5 in half of the SEs, the suggestion that S2 represents a tumour cell with an intermediate phenotype between SE and EC, but not a pure SE (Wenk *et al.*, 1994; von Keitz *et al.*, 1995), is supported.

All SEs (except one) lacked $Le^x$, while this glycolipid was present in all ECs. This marker can thus be used for the differential diagnosis between SE and EC. In primary ECs, the expression of CDH is markedly enhanced, as compared with cell lines. This could mean that CDH is more rapidly converted into the derived globo-series glycolipids in cell lines cultured *in vitro*, especially as the expression of globo-series glycolipids is similar in primary tumours and cell lines.

Results obtained in a NATO advanced study workshop (Andrews *et al.*, 1996) on the expression of cell-surface antigens by TGCT cell lines, applying immunohistochemistry and immunoflow cytometry, largely confirm our data on $Le^x$, detected with antibodies to SSEA-1 (as well as those on SSEA-3 and SSEA-4 expression). However, our data and those presented by Wenk *et al.* (1994) show some differences with those obtained by Andrews *et al.*, (1996). The latter detected SSEA-1 antigen on all cells from the EC cell lines H12.1 and H12.2, whereas Wenk *et al.*, 1994 could not detect this marker on these cell lines, using glycolipid analysis. Most likely, this is due to the fact that although SSEA-1 antigen can be carried on glycolipids, it is mainly presented at the cell surface as glycoprotein (Fenderson *et al.*, 1993). The presented data suggest that this is true for the H12.1 and H12.2 cell lines. However, our results imply that EC cells in primary tumours express the SSEA-1 antigen on the $Le^x$ glycolipid, alone or in addition to expression on glycoproteins (which was not investigated in our study), whereas *in vitro*, this antigen is mainly carried on glycoproteins. Taken together, the studies on cell lines indicate that EC cell lines heterogeneously express SSEA-1 and reduced expression of this marker in cell lines could indicate its loss upon prolonged *in vitro* culture. Our data, those from the NATO workshop and those presented by Wenk *et al.* (1994) implicate the presence in ECs of a large, globo-series glycolipid-expressing stem cell population, which (heterogeneously) expresses SSEA-1, *in vitro* mainly carried on glycoproteins and *in vivo* (also) on glycolipids.

Our data on ECs are not in keeping with those obtained by Motzer *et al.* (1988) and Damjanov *et al.* (1982), who could not immunohistochemically detect SSEA-1 expression in ECs. The use of MAb P12 by Motzer *et al.*, 1988 whereas we used MAb MC480 might account for this difference. The use of MC480 in combination with a two-step detection method by Damjanov *et al.* (1982) might account for their findings, as they also failed to detect SSEA-3 expression in SEs (using the two-step approach), which was detected by us in the present study and in an immunohistochemical analysis using the avidin–biotin method (not shown).

The glycolipid patterns of the two pure YSs, TL1013 and TL37R21, the latter derived from a xenografted NS with a YS component, are similar to those described for YS cell lines. The four primary YSs with minor populations of other non-seminomatous cell types (as indicated in Table II), did not display a clearly defined glycolipid profile. These heterogeneous glycolipid profiles could not be related to the types of tumour morphology distinguished by Pera *et al.* (1987), i.e. solid and reticulated YS resembling rodent visceral and parietal endoderm respectively. In contrast to four previously described YS cell lines (one lacking all detectable glycolipids) (Wenk *et al.*, 1994), these four primary YSs contain $Le^x$. Presence of this glycolipid could probably be attributed to EC or teratoma cells, that were immunohistochemically detected in these YSs as minor cell populations. Damjanov *et al.* (1982) detected $Le^x$ immunohistochemically in the YS cells of tumours containing at least EC and YS components, whereas pure YSs were not analysed. We conclude that pure YSs are characterised by at least lacking Gb5, GL7 and $Le^x$.

Our data on pure ECs and NSs with differentiated components, are in agreement with those reported for the

**Figure 3** Speculative model of the development of testicular germ cell tumours from carcinoma *in situ*, taking into consideration the glycolipid expression patterns of the various tumour types.

cell line NT2 (Wenk *et al.*, 1994). EC cells express almost exclusively large amounts of globo-series glycolipids (apart from $Le^x$). The NSs with differentiated components are characterised by a lower expression of the globo-series glycolipids, especially Gb3 and Gb5, expression of the lacto-series glycolipid $Le^x$ in the majority of the tumours and presence of the gangliosides GD3 and GT3, at the highest levels in tumours with at least an MT component. These data confirm the morphological observations of the presence of a minor stem cell population in differentiated NSs. Although our semi-quantitative analysis of the glycolipid expression in pure tumours indicates which glycolipids are expressed by the various cell types, an immunohistochemical approach could be used to study the distribution of glycolipids, especially concerning the non-seminomatous cell types in mixed tumours.

Our data on the spermatocytic seminoma support the contention that this tumour type is a separate GCT entity, not derived from CIS cells (Burke and Mostofi, 1993; Cummings *et al.*, 1994). Based on their glycolipid content, the spermatocytic seminoma, non-GCTs and the normal parenchyma of the testis could readily be discriminated from TGCTs and parenchyma containing CIS.

In conclusion, our analysis of the glycolipid content of human primary TGCTs confirms the data obtained on non-seminomatous cell lines (Wenk *et al.*, 1994). Globo-series glycolipids are highly expressed in ECs, whereas the expression of especially Gb3 and Gb5 is reduced in differentiated non-seminomatous elements. In addition, we show that the globo-series glycolipids are expressed at similar levels in CIS, SEs and ECs. The expression of $Le^x$ by ECs allows discrimination between this tumour type and SEs, which do not express this marker. Gangliosides are found in many SEs and almost all differentiated NSs, but are rare in ECs. These results could be integrated in the speculative model shown in Figure 3. Primitive cells, i.e. CIS and SE cells, are characterised by globo-series glycolipids. These tumour cells could develop along two pathways. Either they mature (differentiate) in the germ cell lineage and start expressing gangliosides, or they are reprogrammed to become pluripotent EC cells and start expressing lacto-series $Le^x$. When these reprogrammed cells mature (differentiate) into various lineages they start expressing gangliosides as well. The present data fit into the linear progression model, but do not prove it. Studies comparing the glycolipid profile of CIS and adjacent tumour, either SE or NS, should be performed to further investigate this model. In addition, it would be interesting to see if modulation of the glycolipids can change the phenotype of the tumour cells. Preliminary studies with NT2 (EC) cells using the glycosylceramide synthase inhibitor PDMP indicate that glycolipid depletion results in changed growth and shape of the cells (unpublished observations). This issue might also be addressed using transfection with glycosyltransferase genes to change glycolipid patterns. At present, these studies have to be limited to non-seminomatous cell types, as SE cell lines are not available.

**Acknowledgements**
The work described in this report was supported by the Dutch Cancer Society Grant DDHK 91-19 and a Dutch Cancer Society Travel Grant to RAO. Collaborating urologists and pathologists in the south-western part of the Netherlands are thanked for supplying tumour samples. We acknowledge Dr Bloppoel (Department of Chemical Pathology, Erasmus University, Rotterdam) for lyophilisation of the tumour material.

### References

ANDREWS PW, BRONSON DL, BENHAM F, STRICKLAND S AND KNOWLES BB. (1980). A comparative study of eight cell lines derived from human testicular teratocarcinoma. *Int. J. Cancer*, **26**, 269-280.

ANDREWS PW, NUDELMAN E, HAKOMORI S AND FENDERSON BA. (1990). Different patterns of glycolipid antigens are expressed following differentiation of TERA-2 human embryonal carcinoma cells induced by retinoic acid, hexamethylene bisacetamide (HMBA) or bromodeoxyuridine (BUdR). *Differentiation*, **43**, 131-138.

ANDREWS PW, CASPER, J, DAMJANOV, I, DUGGAN-KEEN M, GOWERCMAN A, HATA J-I, VON KEITZ A, LOOIJENGA LHJ, OOSTERHUIS JW, PERA M, SAWADA M, SCHMOLL H-J, SKAKKERBAEK NE, VON PUTTEN W AND STERN P. (1996). A comparative analysis of cell surface antigens expressed by cell lines derived from human germ cell tumors. *Int. J. Cancer*. (in press).

BIRD J AND KIMBER SJ. (1984). Oligosaccharides containing fucose linked α(1-3) and α(1-4) to N-acetylglucosamine cause decompaction of mouse morulae. *Dev. Biol.*, **104**, 449-460.

BREMER EG, HAKOMORI S, BOWEN-POPE DF, RAINES E AND ROSS R. (1984). Ganglioside-mediated modulation of cell growth, growth factor binding, and receptor phosphorylation. *J. Biol. Chem.*, **259**, 6818-6825.

BURKE AP AND MOSTOFI FK. (1993). Spermatocytic seminoma. A clinicopathologic study of 79 cases. *J. Urol. Pathol.*, **1**, 21-32.

CASPER J, SCHMOLL H-J, SCHNAIDT U AND FONATSCH C. (1987). Cell lines of human germinal cancer. *Int. J. Androl.*, **10**, 105-113.

CUELLO AC, GAROFALO L, KENIGSBERG RL AND MAYSINGER D. (1989). Gangliosides potentiate in vivo and in vitro effects of nerve growth factor on central cholinergic neurons. *Proc. Natl Acad. Sci. USA*, **86**, 2056-2060.

CUMMINGS OW, ULBRIGHT TM, EBLE JN AND ROTH LM. (1994). Spermatocytic seminoma: an immunohistochemical study. *Hum. Pathol.*, **25**, 54-59.

CZAJA JT AND ULBRIGHT TM. (1992). Evidence for the transformation of seminoma to yolk sac tumor, with histogenetic considerations. *Am. J. Clin. Pathol.*, **97**, 468-477.

CZERNOBILSKY B. (1991). Differentiation patterns in human testicular germ cell tumours. *Virchows Arch. A Path. Anat. Histol.*, **419**, 77-78.

DAMJANOV I, FOX N, KNOWLES BB, SOLTER, D, LANGE PH AND FRALEY EE. (1982). Immunohistochemical localization of murine stage-specific embryonic antigens in human testicular germ cell tumors. *Am. J. Pathol.*, **108**, 225-230.

DAMJANOV I, HORVAT B AND GIBAS Z. (1993). Retinoic acid-induced differentiation of the developmentally pluripotent human germ cell tumor-derived cell line, NCCIT. *Lab. Invest.*, **68**, 220–232.

DE JONG B, OOSTERHUIS JW, CASTEDO SMMJ, VOS A AND TE MEERMAN GJ. (1990). Pathogenesis of adult testicular germ cell tumors: A cytogenetic model. *Cancer Genet. Cytogenet.*, **48**, 143–167.

DIPPOLD WG, KNUTH A AND MEYER ZUM BUSCHENFELDE K-H. (1984). Inhibition of human melanoma cell growth in vitro by monoclonal anti-GD3-ganglioside antibody. *Cancer Res.*, **44**, 806–810.

EGGENS I, FENDERSON BA, TOYOKUNI T, DEAN B, STROUD MR AND HAKOMORI S. (1989). Specific interaction between Lex and Lex determinants: A possible basis for cell recognition in preimplantation embryos and in embryonal carcinoma cells. *J. Biol. Chem.*, **264**, 9476–9484.

EISENBARTH GS, WALSH FS AND NIRENBERG M. (1979). Monoclonal antibody to a plasma membrane antigen of neurons. *Proc. Natl Acad. Sci. USA*, **76**, 4913–4917.

EL-NAGGAR AK, RO JY, MCLEMORE D, AYALA AG AND BATSAKIS JG. (1992). DNA ploidy in testicular germ cell neoplasms: histogenetic and clinical implications. *Am. J. Surg. Pathol.*, **16**, 611–618.

FENDERSON BA, ZEHAVI U AND HAKOMORI S. (1984). A multivalent lacto-N-fucopentaose III–lysyllysine conjugate decompacts preimplantation mouse embryos, while the free oligosaccharide is ineffective. *J. Exp. Med.*, **160**, 1591–1596.

FENDERSON BA, ANDREWS PW, NUDELMAN E, CLAUSEN H AND HAKOMORI S. (1987). Glycolipid core structure switching from globo to lacto and ganglioseries during retinoic acid-induced differentiation of TERA-2 derived human embryonal carcinoma cells. *Dev. Biol.*, **122**, 21–34.

FENDERSON BA, RADIN N AND ANDREWS PW. (1993). Differentiation antigens of human germ cell tumours: distribution of carbohydrate epitopes on glycolipids and glycoproteins analyzed using PDMP, an inhibitor of glycolipid synthesis. *Eur. Urol.*, **23**, 30–37.

FOGH J AND TREMPE G. (1975). New human tumor cell lines. In: *Human Tumor Cells*, Fogh J. (ed.) pp. 115–159. Plenum: New York.

FOLCH-PI J, ARSOVE S AND MEATH JA. (1951). Isolation of brain strandin, a new type of large molecule tissue component. *J. Biol. Chem.*, **19**, 819–831.

FOSSÅ SD, NESLAND JM, PETTERSEN EO, AMELLEM O, WAEHRE H AND HEIMDAL K. (1991). DNA ploidy in primary testicular cancer. *Br. J. Cancer*, **64**, 948–952.

FRIEDMAN NB. (1951). The comparative morphogenesis of extragenital and gonadal teratoid tumors. *Cancer*, **4**, 265–276.

GILLIS AJM, OOSTERHUIS JW, SCHIPPER MEI, BARTEN EJ, VAN BERLO R, VAN GURP RJHLM, ABRAHAM M, SAUNDERS GF AND LOOIJENGA LHJ. (1994). Origin and biology of a testicular Wilms' tumor. *Genes Chrom. Cancer*, **11**, 126–135.

GOOI HC, FEIZI T, KAPADIA A, KNOWLES BB, SOLTER D AND EVANS MJ. (1981). Stage-specific embryonic antigen involves $\alpha 1 \rightarrow 3$ fucosylated type 2 blood group chains. *Nature*, **292**, 156–158.

HARLOW E AND LANE D. (1988). *Antibodies. A Laboratory Manual*, Cold Spring Harbor Press, Cold Spring Harbor, NY.

IUPAC-IUB. (1978). Commission on Biochemical Nomenclature. *Biochem. J.*, **171**, 21–35.

KANG J-L, RAJPERT-DE MEYTS E, WIELS J AND SKAKKEBÆK NE. (1995). Expression of the glycolipid globotriaosylceramide (Gb3) in testicular carcinoma in situ. *Virchows Arch.*, **426**, 369–374.

KANNAGI R, NUDELMAN E, LEVERY SB AND HAKOMORI S. (1982). A series of human erythrocyte glycosphingolipids reacting to the monoclonal antibody directed to a developmentally regulated antigen, SSEA-1. *J. Biol. Chem.*, **257**, 14865–14874.

KANNAGI R, LEVERY SB, ISHIGAMI F, HAKOMORI S, SHEVINSKY LH, KNOWLES BB AND SOLTER D. (1983*a*). New globoseries glycosphingolipids in human teratocarcinoma reactive with the monoclonal antibody directed to a developmentally regulated antigen, stage-specific embryonic antigen 3. *J. Biol. Chem.*, **258**, 8934–8942.

KANNAGI R, COCHRAN NA, ISHIGAMI F, HAKOMORI S, ANDREWS PW, KNOWLES BB AND SOLTER D. (1983*b*). Stage-specific embryonic antigens (SSEA-3 and -4) are epitopes of a unique globo-series ganglioside isolated from human teratocarcinoma cells. *EMBO J.*, **2**, 2355–2361.

LOOIJENGA LHJ, GILLIS AJM, VAN PUTTEN WLJ AND OOSTERHUIS JW. (1993). In situ numeric analysis of centromeric regions of chromosomes 1, 12, and 15 of seminomas, nonseminomatous germ cell tumors, and carcinoma in situ of human testis. *Lab. Invest.*, **68**, 211–219.

MOSTOFI FK. (1980). Pathology of germ cell tumors of testis. A progress report. *Cancer*, **45**, 1735–1754.

MOSTOFI FK. (1984). Tumour markers and pathology of testicular tumours. In *Progress and Controversies in Oncological Urology*, pp. 69–87. AR, Liss: New York.

MOSTOFI FK, SESTERHENN IA AND DAVIS CJJ. (1987). Immunopathology of germ cell tumors of the testis. *Semin. Diagn. Pathol.*, **4**, 320–341.

MOTZER RJ, REUTER VE, CORDON-CARDO C AND BOSL GJ. (1988). Blood group-related antigens in human germ cell tumors. *Cancer Res.*, **48**, 5342–5347.

OHYAMA C, FUKUSHI Y, SATOH M, SAITOH S, ORIKASA S, NUDELMAN E, STRAUD M AND HAKOMORI S. (1990). Changes in glycolipid expression in human testicular tumor. *Int. J. Cancer*, **45**, 1040–1044.

OHYAMA C, ORIKASA S, SATOH M, SAITO S, OHTANI H AND FUKUSHI Y. (1992). Globotriaosyl ceramide glycolipid in seminoma: Its clinicopathological importance in differentiation from testicular malignant lymphoma. *J. Urol.*, **148**, 72–75.

OLIE RA, LOOIJENGA LHJ, BOERRIGTER L, TOP B, RODENHUIS S, MULDER MP AND OOSTERHUIS JW. (1995*a*). N- and KRAS mutations in human testicular germ cell tumors: incidence and possible biological implications. *Genes Chrom. Cancer*, **12**, 110–116.

OLIE RA, LOOIJENGA LHJ, DEKKER MC, DE JONG FH, DE ROOY DG AND OOSTERHUIS JW. (1995*b*). Heterogeneity in the in vitro survival and proliferation of human seminoma cells. *Br. J. Cancer*, **71**, 13–17.

OLIVER RTD. (1987). HLA phenotype and clinicopathological behaviour of germ cell tumours: possible evidence for clonal evolution from seminomas to nonseminomas. *Int. J. Androl.*, **10**, 85–93.

OLIVER RTD. (1990). Clues from natural history and results of treatment supporting the monoclonal origin of germ cell tumours. *Cancer Surv.*, **9**, 333–368.

OOSTERHUIS JW, DE JONG B, VAN DALEN I, VAN DER MEER I, VISSER M, DE LEIJ L, MESANDER G, COLLARD JG, SCHRAFFORD KOOPS H AND SLEIJFER DM. (1985). Identical chromosome translocations involving the region of the c-myb oncogene in four metastases of a mediastinal teratocarcinoma. *Cancer Genet. Cytogenet.*, **15**, 99–107.

OOSTERHUIS JW AND LOOIJENGA LHJ. (1993). The biology of human germ cell tumours: retrospective speculations and new prospectives. *Eur. Urol.*, **23**, 245–250.

OOSTERHUIS JW, CASTEDO SMMJ, DE JONG B, CORNELISSE CJ, DAM A, SLEIJFER DT AND SCHRAFFORDT KOOPS H. (1989). Ploidy of primary germ cell tumors of the testis. Pathogenetic and clinical relevance. *Lab. Invest.*, **60**, 14–20.

PATTILLO RA, RUCKERT A, HUSSA R, BERNSTEIN R AND DELFS E. (1971). The Jar cell line. Continuous human multihormone production and controls. *In Vitro*, **6**, 398–399.

PERA MF, BLASCO LAFITA MJ AND MILLS J. (1987) Cultured stem-cells from human testicular teratomas: the nature of human embryonal carcinoma, and its comparison with two types of yolk-sac carcinoma. *Int. J. Cancer*, **40**, 334–343.

PIERCE GB AND ABELL MR. (1970). Embryonal carcinoma of the testis. *Pathol. Annu.*, **5**, 27.

SESTERHENN IA. (1985). The role of intratubular malignant germ cells in the histogenesis of germ cell tumors. In *Proceedings of the 2nd Germ Cell Tumor Conference Leeds 8–10 September 1993*, Jones WG, Milford Ward A and Anderson CK. (eds), pp. 25–35, Leeds.

SKAKKEBÆK NE, BERTHELSEN JG, GIWERCMAN A AND MÜLLER J. (1987). Carcinoma-in-situ of the testis: possible origin from gonocytes and precursor of all types of germ cell tumours exept spermatocytoma. *Int. J. Androl.*, **10**, 19–28.

SOLTER D AND KNOWLES BB. (1978). Monoclonal antibody defining a stage-specific mouse embryonic antigen (SSEA-1). *Proc. Natl Acad. Sci. USA*, **75**, 5565–5569.

SVENNERHOLM L. (1964). The gangliosides. *J. Lipid Res.*, **5**, 145–162.

VON KEITZ AT, RIEDMILLER H, NEUMANN K, GUTSCHANK W AND FONATSCH C. (1995). Establishment and characterization of a seminoma cell-line (S2). *Invest. Urol.* (in press).

WENK J, ANDREWS PW, CASPER J, HATA J, PERA MF, VON KEITZ A, DAMJANOV I AND FENDERSON BA. (1994). Glycolipids of germ cell tumors: Extended globo-series glycolipids are a hallmark of human embryonal carcinoma cells. *Int. J. Cancer*, **58**, 108–115.