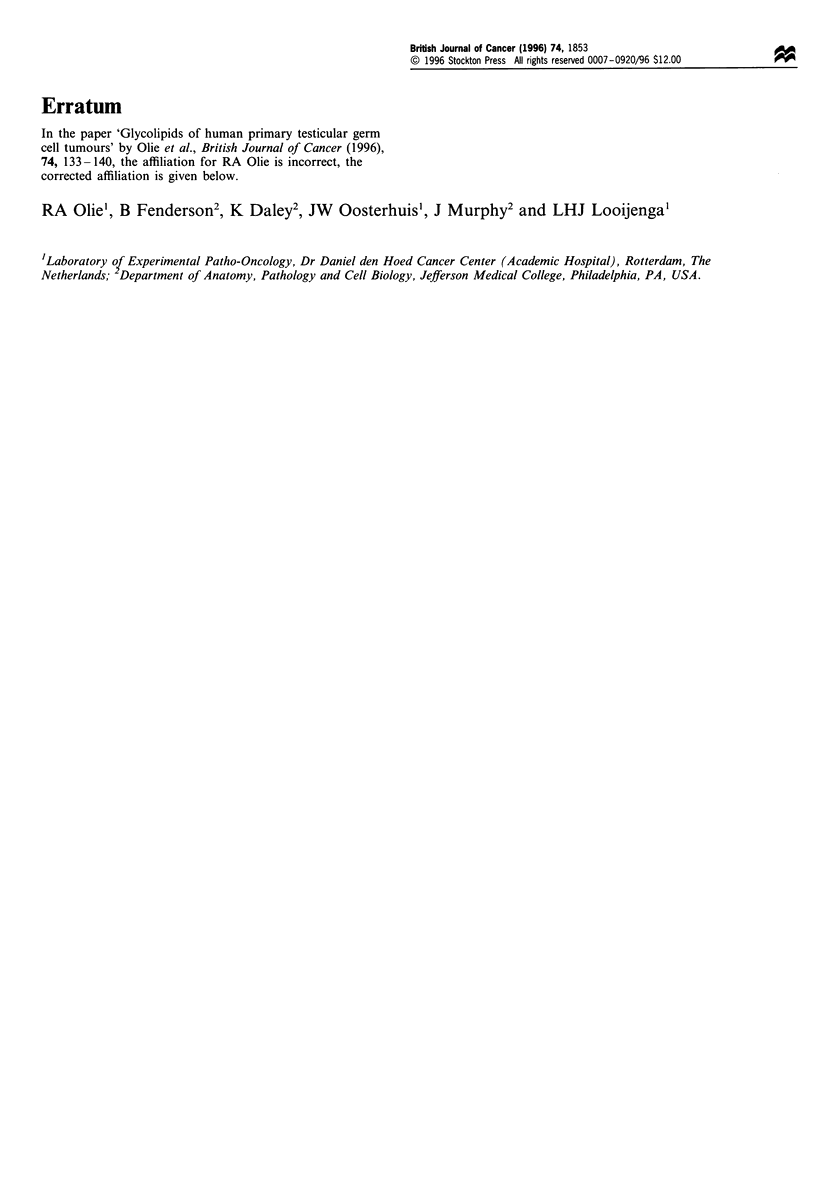# Erratum

**Published:** 1996-12

**Authors:** 


**British Journal of Cancer (1996) 74,** 1853
© 1996 Stockton Press All rights reserved 0007-0920/96 $12.00

### Erratum

In the paper 'Glycolipids of human primary testicular germ cell tumours' by Olie *et al.*, *British Journal of Cancer* (1996), **74,** 133–140, the affiliation for RA Olie is incorrect, the corrected affiliation is given below.

RA Olie[1], B Fenderson[2], K Daley[2], JW Oosterhuis[1], J Murphy[2] and LHJ Looijenga[1]

[1]*Laboratory of Experimental Patho-Oncology, Dr Daniel den Hoed Cancer Center (Academic Hospital), Rotterdam, The Netherlands;* [2]*Department of Anatomy, Pathology and Cell Biology, Jefferson Medical College, Philadelphia, PA, USA.*